# Metabolic consequences of sex reversal in two lizard species: a test of the like-genotype and like-phenotype hypotheses

**DOI:** 10.1242/jeb.245657

**Published:** 2023-07-14

**Authors:** Kristoffer H. Wild, John H. Roe, Lisa Schwanz, Essie Rodgers, Duminda S. B. Dissanayake, Arthur Georges, Stephen D. Sarre, Daniel W. A. Noble

**Affiliations:** ^1^ Division of Ecology and Evolution, Research School of Biology, The Australian National University, Canberra, ACT 2601, AUS; ^2^ Centre for Conservation Ecology and Genomics, Institute for Applied Ecology, University of Canberra, Canberra, ACT 2617, AUS; ^3^ Department of Biology, University of North Carolina Pembroke, Pembroke, NC 28372-1510, USA; ^4^ Evolution and Ecology Research Centre, School of Biological, Earth and Environmental Sciences, University of New South Wales, Sydney, NSW 2052, Australia; ^5^ Centre for Sustainable Aquatic Ecosystems, Harry Butler Institute, Murdoch University, Murdoch, WA 6150, Australia

**Keywords:** Energetics, Sex determination, Sex reversal, *Pogona vitticeps*, *Bassiana duperreyi*

## Abstract

Vertebrate sex is typically determined genetically, but in many ectotherms sex can be determined by genes (genetic sex determination, GSD), temperature (temperature-dependent sex determination, TSD), or interactions between genes and temperature during development. TSD may involve GSD systems with either male or female heterogamety (XX/XY or ZZ/ZW) where temperature overrides chromosomal sex determination to cause a mismatch between genetic sex and phenotypic sex (sex reversal). In these temperature-sensitive lineages, phylogenetic investigations point to recurrent evolutionary shifts between genotypic and temperature-dependent sex determination. These evolutionary transitions in sex determination can occur rapidly if selection favours the reversed sex over the concordant phenotypic sex. To investigate the consequences of sex reversal on offspring phenotypes, we measured two energy-driven traits (metabolism and growth) and 6 month survival in two species of reptile with different patterns of temperature-induced sex reversal. Male sex reversal occurs in *Bassiana duperreyi* when chromosomal females (female XX) develop male phenotypes (male_SR_ XX), while female sex reversal occurs in *Pogona vitticeps* when chromosomal males (male ZZ) develop female phenotypes (female_SR_ ZZ). We show metabolism in male_SR_ XX was like that of male XY; that is, reflective of phenotypic sex and lower than genotypic sex. In contrast, for *Pogona vitticeps*, female_SR_ ZZ metabolism was intermediate between male ZZ and female ZW metabolic rate. For both species, our data indicate that differences in metabolism become more apparent as individuals become larger. Our findings provide some evidence for an energetic advantage from sex reversal in both species but do not exclude energetic processes as a constraint on the distribution of sex reversal in nature.

## Teaser

**Summary:** Analysis of sex reversal in two lizard species indicates that the consequences of energy use and growth vary depending on the phenotype that reverses sex, and energy consequences may differ as individuals grow larger.

## INTRODUCTION

Sex determination in vertebrates is highly variable, ranging from genotypic sex determination (GSD) where sex is established by sex chromosomes, to environmental sex determination (ESD) where sex is primarily influenced by prevailing environmental conditions (Bull, 1980). For some species, these pathways of reproductive development are not mutually exclusive but can co-occur (Bachtrog et al., 2014; Sarre et al., 2004). In a few well-studied species, GSD systems with either male (XX/XY) or female heterogamety (ZZ/ZW) are influenced by incubation temperature (temperature-dependent sex determination, TSD) (Holleley et al., 2015; Noble et al., 2018; Quinn et al., 2009). In these GSD species, conditions experienced during critical developmental stages exceed a threshold temperature that overrides genetic sex-determining mechanisms. This temperature override, commonly referred to as sex reversal, causes a discordance between phenotypic and genotypic sex (Holleley et al., 2015; Quinn et al., 2009; Radder et al., 2009). Theoretical models predict that when sex-reversed individuals have a greater fitness advantage, populations can rapidly lose the heterogametic sex chromosome (XY or ZW) and result in pure TSD lineages within a few generations (Grossen et al., 2011; Holleley et al., 2015; Schwanz et al., 2020). Consequently, these TSD lineages should become widely established in free-living populations where environmental conditions favour their emergence. However, sex reversal in some species is not distributed evenly across ecotypes in natural systems, suggesting free-living animals may experience costs associated with sex reversal that are not accounted for in theoretical models (Bókony et al., 2021; Castelli et al., 2021; Mikó et al., 2021; Wild et al., 2022). Quantifying costs and benefits of sex reversal will help clarify patterns of sex reversal in wild populations and provide insight into the mechanisms that may inhibit or accelerate evolutionary transitions in sex-determining systems (Bachtrog et al., 2014; Sarre et al., 2004).

Of crucial importance for individual growth, reproduction and survival is energy expenditure, which can be estimated by measuring metabolic rates. In both empirical and theoretical studies, estimates for metabolism have been shown to be linked to individual patterns of growth, reproduction and survival (Angilletta, 2009; Peterson et al., 1999; Burton et al., 2011; White et al., 2022). Metabolism (and associated energy expenditure) thus provides a crucial link between individual life-history traits (somatic growth, developmental rates and age at maturity) and population processes (population growth, carrying capacity and rates of competition) in a variety of taxa (Brown et al., 2004; Ernest et al., 2003; Robert Burger et al., 2021; Savage et al., 2004). Importantly, strategies used to secure, allocate and expend energy can vary considerably between phenotypic sexes (Arnqvist et al., 2022; Boratyński et al., 2010; Codding et al., 2011; Geffroy, 2022) and may contribute to energetic differences in sex-reversed individuals and their phenotypic and genotypic counterparts. Exploring how sex reversal impacts metabolism and other traits that relate to energy use will provide insight into observed patterns of sex reversal in natural populations.

**Fig. 1.**
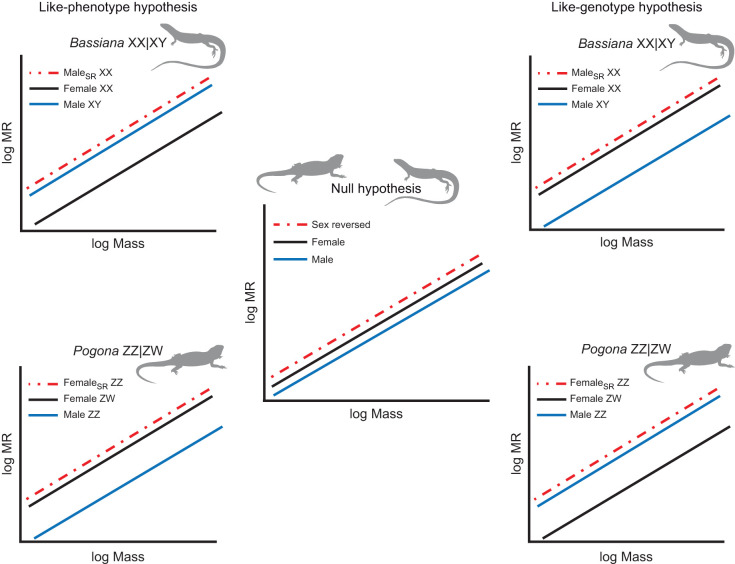
**The like-phenotype/like-genotype framework for testing the metabolic consequences of sex reversal for ZZ/ZW and XX/XY genetic sex determination systems with different patterns of genetic sex determination.** Colours indicate sex class (where SR indicates sex reversed for males and females) for each species (*Bassiana duperreyi* and *Pogona vitticep*). Body mass (g) and metabolic rate (MR; measured as *V̇*_O_2__, ml O_2_ min^−1^) were log transformed to approximate linear relationships. Each pattern of genetic sex determination contains three competing hypotheses for the relationship between body mass and MR: null – no differences; like-phenotype – similarities between reversed sex and concordant phenotype; like-genotype – similarities between reversed sex and concordant genotype.

Here, we tested whether and to what degree sex-reversed individuals differ in metabolism, growth and survival compared with their phenotypic and genotypic counterparts using two species of lizard, *Pogona vitticeps* and *Bassiana duperreyi*, that undergo sex reversal in the wild (Dissanayake et al., 2021a; Holleley et al., 2015; Wild et al., 2022). Sex reversal in *B. duperreyi* occurs when chromosomal females (female XX) develop male phenotypes (male_SR_ XX) (Dissanayake et al., 2021a; Quinn et al., 2009), whereas sex reversal in *P. vitticeps* occurs when chromosomal males (male ZZ) develop female phenotypes (female_SR_ ZZ) (Holleley et al., 2015; Quinn et al., 2007). Three plausible phenotypic/genetic patterns may manifest that can influence the evolution of sex reversal in nature (Fig. 1, e.g. metabolism): (1) there is no difference in metabolism, growth or survival among different genotype–phenotype combinations such that males, females and sex-reversed individuals are indistinguishable (null); (2) sexes are phenotypically similar with discordant sex-reversed individuals (e.g. female_SR_ ZZ or male_SR_ XX) and concordant individuals of the same phenotypic sex (e.g. female ZW, male_SR_ XY) exhibiting similar metabolic rate, growth and/or survival (like-phenotype); or (3) sexes are phenotypically different with discordant sex-reversed individuals (e.g. female_SR_ ZZ or male_SR_ XX) and concordant individuals of the same chromosomal sex (e.g. male ZZ, female XX) exhibiting similar metabolic rate, growth and/or survival (like-genotype).

Evidence for the like-phenotype hypothesis would suggest that metabolic differences between phenotypic sexes (i.e. male versus female) may be driven by hormonal mechanisms or sexually antagonistic selection that leads to sexual dimorphism in traits such as morphology or physiology (Cox et al., 2017; Eyer et al., 2019; Lipinska et al., 2015; van Doorn and Kirkpatrick, 2010). Support for the like-genotype hypothesis would imply that sex-linked genes may be involved in the expression of traits associated with metabolism, energy use and potentially other fitness-related endpoints (Charlesworth and Charlesworth, 1980; Fisher, 1931; Harrison et al., 2015). To date, no studies have explored how energetic components (i.e. metabolism, growth, maintenance) are affected by sex reversal, even though sex-specific strategies of energy allocation have been documented between phenotypic males and phenotypic females (Geffroy, 2022; Somjee et al., 2022).

## MATERIALS AND METHODS

### Lizard collection and husbandry

Field trips and respirometry collection were performed under ANU Animal Ethics approval AEEC A2019-41 and University of Canberra Animal Ethics approval AEC 17-13.

#### Bassiana duperreyi

Twenty-five *Bassiana duperreyi* (Gray 1838) nests with a total of 40 eggs (1–4 eggs per nest) were opportunistically located in November 2020 by flipping rocks, logs and other cover objects at two field locations within the Brindabella Range (Mount Ginini: 1640 m a.s.l., 35°31′29.6″S 148°46′58.7″E; Piccadilly Circus: 1240 m a.s.l., 35°21′42.0″S 148°48′12.5″E). These sites were selected because of high frequencies of sex reversal previously documented within these populations (Dissanayake et al., 2021a). The number of eggs per nest was recorded, and temperature dataloggers (iButton^®^ model DS1921G; accuracy ±1°C) were placed at the core of each nest to monitor nest temperatures. Each nest was maintained in natural conditions for 9–10 weeks at each location, and the mean nest temperatures (Mount Ginini: 18.94±0.98°C; Piccadilly Circus: 20.42±0.84°C; Fig. S1) were monitored to ensure approximately 90% of the development period passed in natural conditions (Shine et al., 2002). Therefore, sex reversal in *B. duperreyi* occurred in natural nest sites as a result of exposure to sex-reversing low temperatures (<20°C) *in situ*. The eggs were then collected, placed in moist vermiculite, and transported back to the University of Canberra. Eggs were placed in incubators (LabWit, ZXSDR1090) that maintained 23°C, which produces a balanced sex ratio (Shine et al., 2002). For the study site description and further details regarding general egg collection methods, see Dissanayake et al. (2021b).

Phenotypic sex was determined by squeezing the tail base to evert the hemipenes (Harolow, 1996) for 3–7 day old hatchlings and was checked again by hemipene transillumination after 5 weeks (Dissanayake et al., 2021b). Blood from the tail of each individual was collected on Whatman FTATM Elute Micro Card (cat no. WB120410). Lizards were housed individually in plastic containers (0.35×0.25×0.15 m). Each tub contained cardboard tubes and paper egg carton pieces for hides. Full-spectrum UV bulbs and heat bulbs were placed alternating between tubs to create a thermal gradient in each tub (heat from one side, UV from the other). Hatchlings were fed live, gut-loaded crickets once per day *ad libitum*, and twice per week the crickets were dusted with calcium powder. Hatchlings were provided with shallow water dishes that were replenished daily, and they were misted twice per day with water.

#### Pogona vitticeps

The University of Canberra maintains a breeding colony of adult *Pogona vitticeps* Ahl 1927, where breeding enclosures are composed of one male (male ZZ) to either three sex-reversed females (female_SR_ ZZ) or three concordant females (female ZW). During the summer of 2020–2021, females were allowed to lay eggs, which were collected within 2 h of deposition. Eggs (*n*=96) from 15 clutches were randomly allocated to either 28°C (*n*=43; no sex reversal expected) or 34°C (*n*=53; reversal of 50% of ZZ genotypes expected) in temperature-controlled incubators (LabWit, ZXSDR1090) on moist vermiculite. Thus, sex reversal in *P. vitticeps* occurred because of exposure to sex-reversing high temperatures (>32°C) during incubation. Once hatchlings emerged, the determination of phenotypic sex and blood sampling followed the same protocols as for *B. duperreyi*. Hatchlings were housed in plastic tubs (0.8×0.5×0.35 m; 5 individuals per tub), and in addition to crickets, finely grated vegetables were introduced to the diet beginning at 6–7 weeks post-hatch.

### Genotyping

Genotypic sex was determined for both *B. duperreyi* and *P. vitticeps* using PCR-based molecular sex tests from extracted DNA collected from tissue samples (Dissanayake et al., 2020; Holleley et al., 2015). DNA purity was determined using a NanoDrop 1000 spectrophotometer (NanoDrop Technologies Inc., Wilmington, DE, USA) and quantified using a Qubit 2.0 Fluorometer (Invitrogen, Life Technologies, Sydney, NSW, Australia). The sex reversal status was determined for *B. duperreyi* by using PCR as described by Dissanayake et al. (2020), where the genotypic sex was identified based on Y-specific markers allowing identification of XX and XY samples. No XY females were observed, which is consistent with previous observations that recombination and/or mutation involving these loci is negligible and does not affect the accuracy of genotypic sex assignment. Genotypic sex (ZZ/ZW) for *P. vitticeps* was determined using a PCR-based molecular sex test that amplifies a W chromosome-specific size polymorphism (Holleley et al., 2015), in which two bands amplify in ZW individuals and one control band amplifies in ZZ individuals. No ZW males were observed. All PCR products were visualized on a 1.5% agarose gel using SYBR Safe (Life Technologies, Carlsbad, CA, USA), and all molecular sex tests were conducted blind to the phenotypic sex of the individuals. For both species, sex class accounted for genotype and phenotype and when genotype–phenotype discordance occurred, individuals were classified as sex reversed (Holleley et al., 2015).

### Respirometry

Metabolic rate (MR) was defined as the rate of oxygen consumption *V̇*_O_2__, ml min^−1^) of the post-absorptive animal using a stop flow respirometry system (Sable Systems FMS, Las Vegas, NV, USA). A gas analyser sub-sampler was used to pump outside air scrubbed of CO_2_ (using soda lime, ChemSupply Australia, Gillman, SA, Australia) and water vapour (using Drierite, W. A. Hammond Drierite Co. Ltd) to a mass flow controller that regulated flow rate to a nominal value of 130 ml min^−1^ (*B. duperreyi*) or 250 ml min^−1^ (*P. vitticeps*). After passing through the mass flow controller, air was pushed through an airtight cylindrical respirometry chamber, with dimensions designed specifically for each species (*B. duperreyi*: 75×20 mm; *P. vitticeps*: 200×40 mm). Air was pushed into the chamber and then through a flow meter, ensuring that flow rates were constant. Air was then rescrubbed of water vapor, using Drierite, before being passed through H_2_O and O_2_ gas analysers. The fractional concentration of O_2_ in the ex-current air (*F*e_O_2__) was recorded at a frequency of 1 Hz. Following the manufacturer's protocols, both H_2_O and O_2_ analysers were calibrated prior to experiments.

MR was measured within 3 weeks of hatching for all individuals. After a minimum fasting period of 24 h, body mass (0.01 g) was measured of each individual lizard using a digital scale (Ohaus SP-202) before and after it was placed in the respirometry chamber. In the respirometry experiments, we utilized two incubators (LabWit, ZXSDR1090). The first incubator controlled the temperature of the exterior air, which was then directed into the second incubator. This second incubator controlled the temperature (±1°C) of the respirometry chambers placed inside it. Incubator temperatures were held at a constant temperature relevant to the thermal preference for each species [*B. duperreyi* 34°C (Du et al., 2010); *P. vitticeps* 33°C (Greer, 1989)]. At approximately 17:00 h, lizards were placed in respirometry chambers inside a dark incubator and remained in the chambers overnight for the duration of the experiment. As such, these animals were mainly in a quiescent state, but some activity may have occurred within the chamber. Each respirometry trial lasted approximately 14 h, and to ensure that animals were habituated within chambers, the first 2 h of data were discarded from analysis. The system contained seven chambers that lizards were placed in individually and one empty chamber designated as a control. The O_2_ consumption of each lizard was measured continuously for 5 min over a 70 min sampling window, and this sampling sequence was repeated every 70 min for the duration of the experiment. Immediately following each individual lizard measurement, the control chamber was recorded for 5 min as a baseline of O_2_. During each 70 min sampling window, O_2_ depletion for each individual was identified using the R package ‘metabR’ (github.com/daniel1noble/metabR). The rate of O_2_ (*V̇*_O_2__, ml min^−1^) depleted by an individual was calculated following eqn 4.21 in Lighton (2008):
(1)

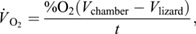
where the %O_2_ is the maximum percentage of O_2_ contained in a sample below the baseline; *V*_chamber_ is the volume of the chamber (*B. duperreyi*: 23.56 ml; *P. vitticeps*: 251.33 ml); *V*_lizard_ is an average between the pre- and post-measurement mass of each individual; and *t* is the duration of time the chamber was sealed between air samples (70 min). The mass of each lizard was used as a proxy for its volume (1 g=1 ml) because of their high correlation and increased accuracy and precision in mass measurements (Friesen et al., 2017).

### Growth and survival

Measurements of snout–vent length (SVL) and mass were used to estimate growth rates. SVL and mass were initially measured during respirometry experiments and remeasured 6 months after the initial measurements. Growth rate was calculated by subtracting initial measurements (SVL or mass) from the final remeasurement and dividing by the elapsed time between measurements. SVL growth rate was recorded in mm day^−1^ for both species, and mass growth rate was recorded in cg day^−1^ for *B. duperreyi* and g day^−1^ for *P. vitticeps*. The survival rate of hatchlings was determined by documenting the frequency of mortality between the hatch date and 6 months post-hatch date for both species.

### Statistical analysis

All statistical analyses were conducted using the R environment, v. 4.1.0 (www.r.-project.org). Bayesian linear mixed effect models from the package ‘brms’ (Bürkner, 2017) were used to analyse O_2_ data for each species. We used Bayesian modelling approaches because of their flexibility with respect to parameter estimation. It is also easier to interpret and manipulate posterior probabilities for each parameter in the model. Default priors (see Supplementary Materials and Methods for details) were used and four MCMC chains of 5000 were run with a burn in of 1000 and a thinning interval of 5 for the brms models. All models were checked for proper mixing and convergence by visually inspecting trace plots. For each species, two models were fitted, the first in which homoscedasticity of the data was assumed and the second in which heteroscedasticity was accounted for within the data. The first model for estimating metabolism was fitted using the following structure:
(2)

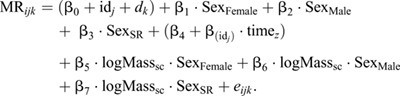
MR*_ijk_* is the metabolic rate (log*V̇*_O_2__) for measurement *i* (*i*=1 to *N*_m_, number of measurements) on individual *j* (*j*=1 to *N*_id_, number of individuals) and day *k* (*k*=1 to *N_d_*, number of days). β_1_−β_3_ are contrasts for the different sex classes, where Sex_female_ and Sex_­male_ are for concordant sexes and Sex_SR_ is for sex-reversed animals, respectively. A linear slope β_4_ was estimated for measurement time (time*_z_*, *z*-transformed) and a random intercept (id*_j_*) and slope for time*_z_* (β_id*_j_*_) were included for individual *j* across measurement occasions. A linear slope for log-transformed mass (logMass_sc_, centred on mean, sc) and mass scaling relationships were estimated separately for the different sex classes (i.e. β_5_·logMass_sc_·Sex_Female_, β_6_·logMass_sc_·Sex_Male_ and β_7_·logMass_sc_·Sex_SR_, respectively). Deviations were sampled from a multivariate normal distribution (∼MVN[0,0],ID), where ID is a (co)variance matrix with a random intercept and slope variance and their covariance. A random-effect for day (*d_k_*) [∼*N*(0,σ^2^*_k_*)] was also included in the model to account for variation across days in MR. In all models, we retained data for each measurement throughout the night to improve analytical power. Given that animals were quiescent, our MR data are expected to be representative of standard metabolic rate (SMR). Nonetheless, some movement did occur in our chambers. As such, we also fitted the same models described above but kept the lowest 10% of oxygen consumption values during trials – data that should be quite close to SMR. We found no changes in our results when using the full dataset compared with the dataset that only used the lowest 10% (see Fig. S2, Tables S1 and S2). Therefore, all *V̇*_O_2__ measurements from trials (MR) were kept for further analysis.

Differences in growth rates were compared across sex class using Bayesian linear models while accounting for individual mean metabolism. This allowed us to test whether there was a relationship between metabolism and growth rate (mass or SVL) across sex class. Fisher's exact tests were used to determine whether there was an association between sex class and frequency of hatchling mortality after 6 months.

For all Bayesian models, posterior estimates were from four MCMC chains, and we present posterior means and their 95% credible intervals (CIs). For each species, an additional model was fitted that accounted for heteroscedasticity within the data, and leave-one-out (loo) cross-validation was used to compare the predictive accuracy between the homoscedastic and heteroscedastic model. To test for the like-genotype (genotype – sex reversed) or like-phenotype (phenotype – sex reversed) framework for each species, contrasts were calculated by subtracting the posterior distributions of each sex class. To test whether the magnitude of these differences varied significantly, probabilities of parameter estimates were considered statistically significant when the 95% CIs did not include 0, and the pMCMC values were less than 0.05. Data, code and additional resources are available at: https://github.com/daniel1noble/energy_sex_reversal.

## RESULTS

### Energetic consequences of sex reversal

#### Bassiana duperreyi

**Fig. 2.**
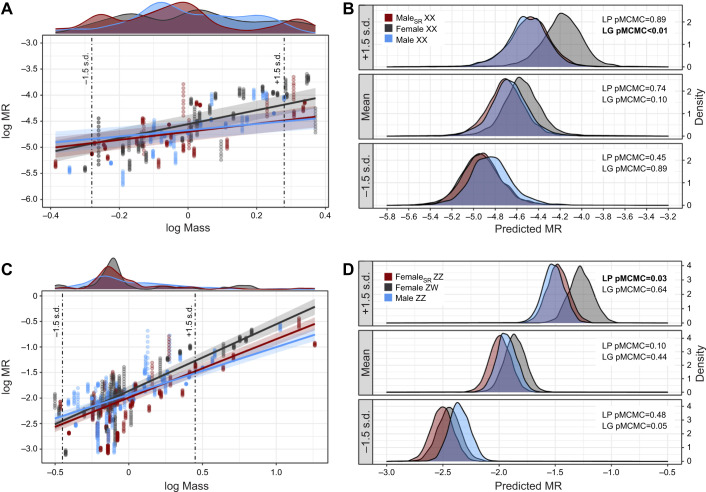
**Comparison of log MR across log mass by sex class for *B. duperreyi* and *P. vitticep*.** MR (ml O_2_ min^−1^) and body mass (g) were measured for *n*=40 *B. duperreyi* (A,B) and *n*=96 *P. vitticeps* (C,D). Sex-reversed individuals (male_SR_ XX or female_SR_ ZZ) are denoted by red, phenotypic females (female XX or female ZW) are denoted by black, phenotypic males (male XY or male ZZ) are denoted by blue. Fitted lines were obtained from predicted values from the brms model for each species and confidence bands were constructed from the s.e. of prediction values for each sex (A,C). Density plots above each regression plot denote the distribution in body mass (log mass) by sex for each species. To visualize how log MR changes across log mass, B and D show the distribution of predicted MR at three areas of log mass (mean±1.5 s.d.) denoted by the dash-dot line in A and C for each sex and species. In A and C, pMCMC values indicate contrast differences between like-phenotype (LP) or like-genotype (LG) for each distribution, and bold indicates a significant pMCMC effect (pMCMC<0.05). Details for these comparisons can be found in Table 4.

A total of 760 measurements for 40 individuals (male_SR_ XX: *n*=13, female XX: *n*=15, male XY: *n*=12) were recorded. There was a strong scaling relationship between log MR and log mass (Table 1), and scaling slopes varied significantly depending on sex class (significant interaction of sex class×log mass; Fig. 2A). Male_SR_ XX had a mass-specific MR that was most like that of their phenotypic counterparts (male_SR_ XX – male XY; pMCMC=0.33; Table 3) and lower than that of their genotypic counterparts (male_SR_ XX – female XX; pMCMC<0.01). For phenotypic males (male_SR_ XX and male XX), the scaling relationship between log mass and metabolism changed similarly across differently sized individuals (Fig. 2B, Table 4). Pairwise comparisons across sex class indicated no differences in body mass across our treatments (Fig. 2A; Table S3). The homogeneous variance model was the most parsimonious [(heteroscedastic model−homoscedastic model) loo: −5.5, s.e.=6.87], accounting for 77% (95% CI: 0.75–0.78) of the variation in MR.

**Table 1.**
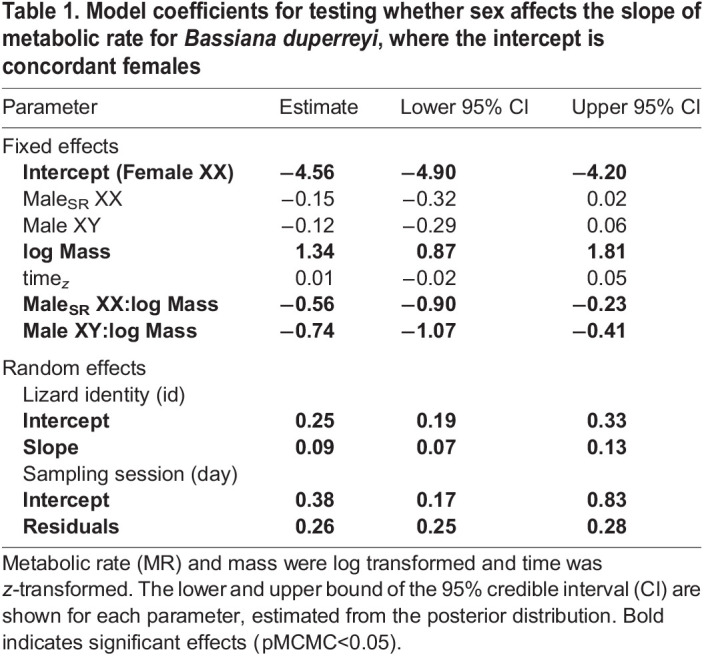
Model coefficients for testing whether sex affects the slope of metabolic rate for *Bassiana duperreyi*, where the intercept is concordant females

#### Pogona vitticeps

A total of 1365 measurements for 96 individuals (female_SR_ ZZ: *n*=28, female ZW: *n*=30, male ZZ: *n*=38) were recorded. There was a strong scaling relationship between log MR and log mass (Table 2), and scaling slopes varied significantly depending sex class (significant interaction of sex class×log mass; Fig. 2C). Sex-reversed female *P. vitticeps* (female_SR_ ZZ) had a mass-specific MR that was overall higher than that of their genotypic counterparts (female_SR_ ZZ – male ZZ; pMCMC<0.01), but lower than that of their phenotypic counterparts (female_SR_ ZZ – female ZW; pMCMC=0.04; Table 3). The mass scaling relationship of metabolism for female_SR_ ZZ was more like that of ZZ males than ZW females (Fig. 2D, Table 4). As a consequence, large female­_SR_ ZZ have significantly lower metabolism compared with female ZW of comparable size (see Fig. 2D, Table 4). Pairwise comparisons of body mass across sex class in *P. vitticeps* indicated no differences in body mass across treatments (Fig. 2C; Table S3). The heteroscedasticity variance model was the most parsimonious [(heteroscedastic model – homoscedastic model) loo: −189.8, s.e.=33.96], accounting for 84% (95% CI: 0.83–0.85) of the variation in MR.

**Table 2.**
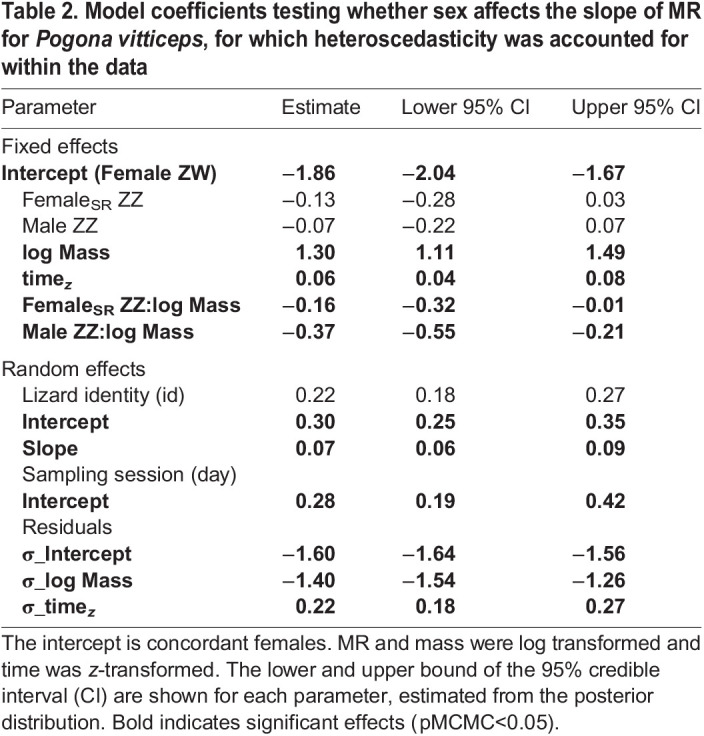
Model coefficients testing whether sex affects the slope of MR for *Pogona vitticeps*, for which heteroscedasticity was accounted for within the data

**Table 3.**
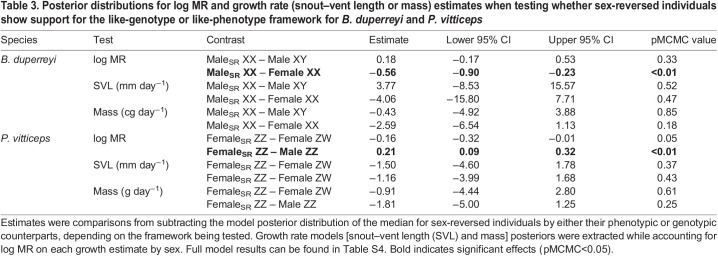
Posterior distributions for log MR and growth rate (snout–vent length or mass) estimates when testing whether sex-reversed individuals show support for the like-genotype or like-phenotype framework for *B. duperreyi* and *P. vitticeps*

**Table 4.**
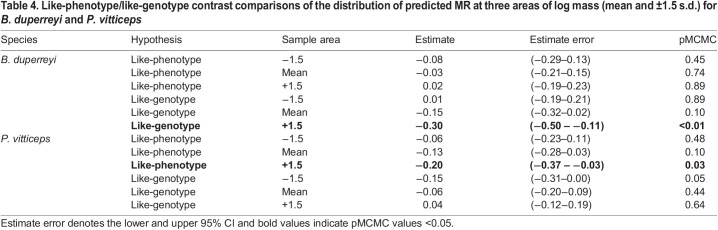
Like-phenotype/like-genotype contrast comparisons of the distribution of predicted MR at three areas of log mass (mean and ±1.5 s.d.) for *B. duperreyi* and *P. vitticeps*

### Effects of sex reversal on growth and survival

Growth rates for both SVL and mass supported the null prediction for *B. duperreyi*, where there were no detectible differences across sex class (Table 3). Similarly, in *P. vitticeps*, the null prediction was supported when comparing SVL and mass growth rates across sex class (Table 3). For both species, there was no relationship between metabolism and growth rate estimates (Table S4). Sex-reversed male *B. duperreyi* had the lowest rates of survival (77%; Table 5) in comparison to concordant females (87%) and concordant males (100%), but this relationship was non-significant (*P*=0.29). Similarly, sex-reversed *P. vitticeps* individuals had the lowest rates of survival (75%; Table 5) in comparison to concordant females (83%) and concordant males (95%), but this relationship was also not significant (*P*=0.06).

**Table 5.**
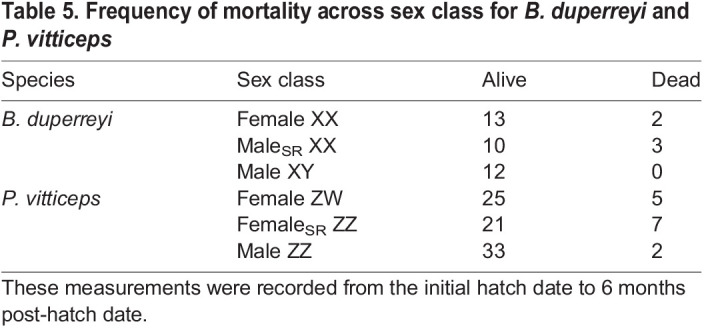
Frequency of mortality across sex class for *B. duperreyi* and *P. vitticeps*

## DISCUSSION

We examined two species of lizard with different modes of sex reversal to test whether metabolism, growth and survival differed between sex-reversed individuals and others of the same phenotypic and genotypic sex. Metabolic responses differed between the two species, with clear support for the like-phenotype hypothesis when males reverse sex (male_SR_ XX; *B. duperreyi*) and equivocal support for each hypothesis when females reverse sex (female_SR_ ZZ; *P. vitticeps*). For both species, regardless of whether individuals reversed sex, phenotypic females required more energy than phenotypic males as individuals grew larger. While sex-reversed animals appeared to have reduced survival, albeit not significantly so, there was no clear evidence in either species for growth advantages over their phenotypic sex. Together, our results suggest that traits associated with energy use and growth may not be strongly tied to genes on the sex chromosomes. Other mechanisms, such as hormonal pathways or differences in immune function, may better explain the stronger signal for phenotypic sex differences (Cox et al., 2017; Kelly et al., 2018; van Doorn and Kirkpatrick, 2010). Assuming similar patterns occur in natural populations, energetic processes may have varying impacts on the species' life-history traits, which could provide insight into what constrains the distribution of sex reversal in nature.

Regardless of the sex-determining system, we show that females had higher mass scaling relationships for metabolism than males (Tables 1 and 2). Hormone-mediated effects, such as responses to elevated levels of thyroxin or corticosterone, have been shown to be responsible for increasing MR for female lizards, and these same hormones are important regulators of phenotypic sex differences in adults (DuRant et al., 2008; John-Alder, 1990; Meylan et al., 2010). Such differences in hormonal pathways may be responsible for the observed concordant sex differences in metabolism, but hormonal responses may transpire differently depending on the phenotype that undergoes sex reversal. However, how endogenous hormone levels shift during early ontogeny for male and female lizards remains poorly understood (but see Lovern et al., 2001) and requires further attention when accounting for sex-reversed individuals as they mature.

We showed that metabolic scaling relationships of sex-reversed individuals differed depending on the GSD system. In the ZZ/ZW system of *P. vitticeps*, larger sex-reversed females (female_SR_ ZZ; >+1.5 s.d. above mean mass) had lower metabolism (15%) than concordant females (female ZW) of similar size (Fig. 2D, Table 4), whereas we observed no such differences for small-sized hatchlings. Given that selection for larger hatchling lizards in the wild is common in lizards (i.e. ‘bigger is better’ hypothesis; Ferguson and Fox, 1984; Sinervo et al., 1992; Warner and Andrews, 2002), this would imply energetic differences between adult sex-reversed and concordant female *P. vitticeps*. As such, we predict that adult female_SR_ ZZ may have more residual energy than female ZW to allocate towards storage, production or activity after resting metabolic costs have been paid. Such surplus in energy reserves for female_SR_ ZZ may explain why sub-adult (<1 year) and adult female_SR_ ZZ *P. vitticeps* are more similar to male ZZ in behaviour and morphology, including higher activity, levels of aggression, and larger body size in captivity (Holleley et al., 2015; Li et al., 2016). However, further work is needed to investigate whether these different strategies of energy allocation exist and how they translate to the observed differences between phenotypic females in body mass, body size and fecundity in wild populations of *P. vitticeps* (Wild et al., 2022). Given that our results indicate that the magnitude of metabolic differences varies across sexes as individuals get larger (Fig. 2), investigating ontogenetic changes associated with sex reversal will provide promising insights into the consequences of such effects.

In contrast to *P. vitticeps, B. duperreyi* showed strong support for the like-phenotype hypothesis. One simple explanation for this finding is that traits linked to metabolism are of little or no consequence for males. Alternatively, traits linked to metabolism for sex-reversed males (male_SR_ XX) in this species may not be associated with sex chromosomes and are linked to hormonal levels relevant to the phenotypic sex. This hypothesis is plausible if phenotypic males share similarities in their gonadal steroid levels, specifically testosterone. If this hypothesis is correct, then it is likely that steroid levels would have a comparable effect on their metabolism compared with females, and the strength of these signals could differ across life stages or seasons (Marler and Moore, 1989; Oppliger et al., 2004; Zena et al., 2019). Some support for this idea exists in *Anolis carolinensis*. Plasma testosterone concentrations in males are upwards of 4 times higher than in similar-sized females 2 weeks post-hatch, and this difference in testosterone persists throughout juvenile growth where male testosterone can be 3–10 times higher than in females (Lovern et al., 2001). If these hormonal differences were to exist between phenotypes in *B. duperreyi*, this may provide a mechanism for why male_SR_ XX are more like their phenotypic sex.

Overall, there has been little attention focused on how growth or survival differs in sex-reversed individuals compared with their phenotypic or genotypic sex. While we did not detect a significant difference in growth or survival, in both species, sex-reversed hatchlings had a higher frequency of mortality over a 6 month period than the other sexes. High mortality has been previously observed in sex-reversed individuals in laboratory experiments (Mikó et al., 2021) and in the wild (Wild et al., 2022). The lack of clear evidence for differences in metabolism, growth and survival for sex-reversed individuals (male_SR_ XX or female_SR_ ZZ) over their concordant phenotypic sex (male XY or female ZW) in our study provides insight into the factors that may explain the occurrence of sex reversal in the wild. While egg incubation differed between the species for logistical reasons – for *B. duperreyi*, 90% occurred in the field, while in *P. vitticeps*, all eggs were incubated in the laboratory – we do not expect this to impact the relative differences we observed between sex-reversed and concordant individuals in these two species. In both species, incubation temperatures mimicked nest temperatures documented in the wild (Castelli et al., 2021; Dissanayake et al., 2021b), and all hatchlings were reared under common laboratory conditions for the first 6 months of life when all measurements were taken. Further investigation is required to understand the cause of this low survivorship and the demographic consequences these results have for the emergence of sex reversal (Cotton and Wedekind, 2009). Overall, the lack of explicit support in our data for the like-genotype hypothesis in metabolism, growth or survivorship reveals clues to the mechanisms that drive sex reversal in nature.

## Supplementary Material

10.1242/jexbio.245657_sup1 Supplementary information
