# Supplementary material for: Metabolic consequences of sex reversal in two lizard species: a test of the like-genotype and like-phenotype hypotheses
Source: J Exp Biol. 2023 Jul 14;226(13):jeb245657. doi: 10.1242/jeb.245657 (PMC10357012; doi:10.1242/jeb.245657)
Supplement: Supplementary information [file jexbio-226-245657-s1.pdf]

## Supplementary Materials and Methods

Default priors for all Bayesian models were used. For all population-level (i.e. Fixed effects), the default prior for the intercept is a normal distribution with a mean 0 and standard deviation 10. The default prior for the shape parameter of the intercept was a Student-t distribution with mean 0, scale 2.5, and 3 degrees of freedom. The default prior for residuals (sigma) was a Student-t distribution with mean 0, scale 2.5, and 3 degrees of freedom. The Cholesky factor was used as the default prior for correlations between random effects.

To determine if standard metabolic rate (SMR) resulted in different conclusions compared to if we used all metabolic measurements taken over night, we refit our models using only SMR. We defined SMR as the lowest 10% of values of oxygen consumption rate during our overnight trials. For both species this resulted in the removal of nearly 90% of our data and resulted in higher sampling error (unsurprisingly). Nonetheless, this did not change the overall results. Below we provide the detailed results and corresponding figures and tables using SMR for each species.

*Bassiana duperreyi* - Once SMR data (lowest 10% of metabolic rate) were removed, we had a total of 83 measurements for 40 individuals ($male_{SR}$ XX: *n = 13*, female XX: *n = 15*, male XY: *n = 12*). There was a strong scaling relationship between log metabolic rate and log mass (Table S1). Sex-reversed male XX *B. duperreyi* had a scaling relationship that was most like their phenotypic counterparts (male XY - $male_{SR}$ XX; pMCMC = 0.26; Table S1; Fig. S1) compared to their genotypic counterparts (female XX - $male_{SR}$ XX; pMCMC = 0.07). The homogeneous variance model was the most parsimonious ([heteroscedastic model – homoscedastic model] loo: -1.31, SE = 2), accounting for 73% (95% CI:0.63 - 0.8) of the variation in metabolic rate.

*Pogona vitticeps* - Once SMR data (lowest 10% of metabolic rate) were filtered we had a total of total of 146 measurements for 96 individuals ($female_{SR}$ ZZ: *n = 28*, female ZW: *n = 30*, male ZZ: *n = 38*) were recorded. There was a strong scaling relationship between log metabolic rate and log mass (Table S2). Sex-reversed female *P. vitticeps* ($female_{SR}$ ZZ) had a scaling relationship that was overall higher than their genotypic counterparts (male ZZ - $female_{SR}$ ZZ; pMCMC = 0.61; Fig S1), but lower than their phenotypic counterparts (female ZW - $female_{SR}$ ZZ; pMCMC = 0.64; Table 2).The heteroscedasticity variance model was the most parsimonious ([heteroscedastic model – homoscedastic model] loo: -16.4, SE = 5.03), accounting for 86% (95% CI:0.79 - 0.92) of the variation in metabolic rate.

Journal of Experimental Biology • Supplementary information

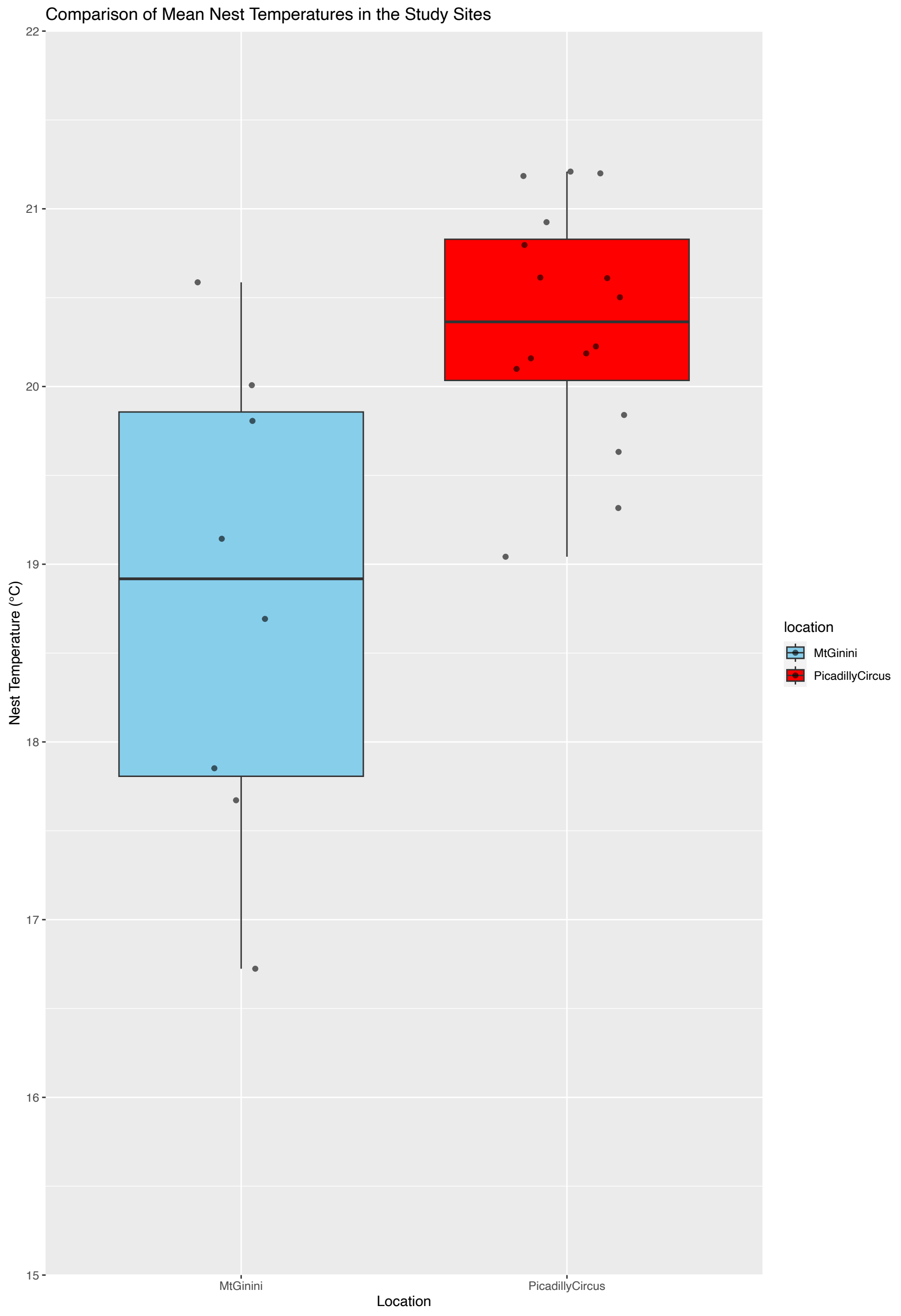


**Fig. S1.** Comparison of mean nest temperatures between Piccadilly Circus and Mt Ginini.

Journal of Experimental Biology • Supplementary information

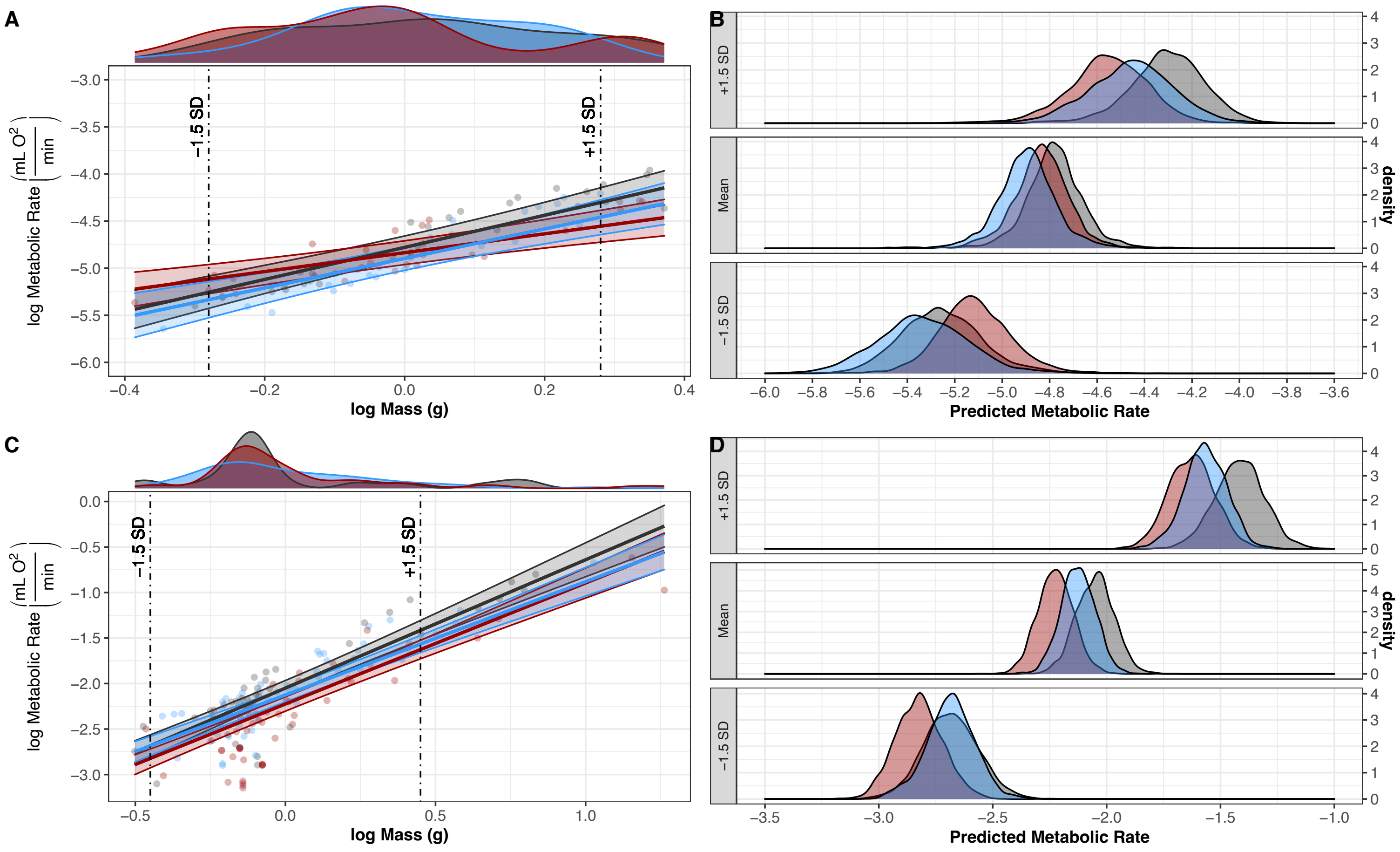


**Fig. S2.** Comparison of log standard metabolic rate (V O2 mL min$^{-1}$) across log body mass (g) by sex class for *Bassiana duperreyi* (A-B) and *Pogona vitticeps* (C-D). Sex-reversed individuals (male$_{SR}$ XX or female$_{SR}$ ZZ) are denoted by red colour, phenotypic females (female XX or female ZW) are denoted in black, phenotypic males (male XY or male ZZ) are denoted in blue. Fitted lines were obtained from predicted values from the brms model for each species and confidence bands were constructed from the SE of prediction values for each sex (A,C). Density plots above each regression plot denote the distribution in body mass (log mass) by sex for each species. To visualize how log metabolic rate changes across log body mass, panels (B and D) show the distribution of predicted metabolic rate at three areas of log body mass (mean, +1.5SD, and -1.5SD) denoted by the dash-dot line in panels A and C for each sex and species

**Table S1.** Model coefficients for testing whether sex affects the slope of standard metabolic rate for *Bassiana duperreyi*, where the intercept is concordant females. Metabolic rate and mass were log transformed and time was z-transformed. Columns l-95% CI and u-95% CI, are the lower and upper bound of the 95% credible interval for each parameter, estimated from the posterior distribution.

| Parameter | Estimate | l-95% CI | u-95% CI |
|---|---|---|---|
| *Fixed Effects* | | | |
| **Intercept (FemaleXX)** | **-4.78** | **-5.02** | **-4.52** |
| Male$_{SR}$XX | -0.06 | -0.22 | 0.10 |
| MaleXY | -0.12 | -0.28 | 0.05 |
| **logMass** | **1.70** | **0.93** | **2.52** |
| ztime | 0.01 | -0.06 | 0.08 |
| Male$_{SR}$XX:logMass | -0.70 | -1.52 | 0.08 |
| MaleXY:logMass | -0.14 | -1.09 | 0.85 |
| *Random Effects* | | | |
| Lizard Identity (id) | | | |
| Intercept | 0.08 | 0.00 | 0.19 |
| Slope | 0.07 | 0.00 | 0.18 |
| Sampling Session (day) | | | |
| **Intercept** | **0.38** | **0.17** | **0.83** |
| **Residuals** | **0.26** | **0.21** | **0.32** |

Journal of Experimental Biology • Supplementary information

**Table S2.** Model coefficients form hetero testing whether sex affects the slope of metabolic rate for *Pogona vitticeps,* which heteroscedasticity was accounted for within the data. The intercept is concordant females. Metabolic rate and mass were log transformed and time was z-transformed. Columns l-95% CI and u-95% CI, are the lower and upper bound of the 95% credible interval for each parameter, estimated from the posterior distribution.

| Parameter | Estimate | l-95% CI | u-95% CI |
|---|---|---|---|
| *Fixed Effects* | | | |
| **Intercept (FemaleZW)** | **-2.05** | **-2.22** | **-1.89** |
| Female$_{SR}$ZZ | -0.17 | -0.34 | 0.02 |
| MaleZZ | -0.08 | -0.22 | 0.06 |
| **logMass** | **1.41** | **1.08** | **1.76** |
| ztime | 0.00 | -0.07 | 0.07 |
| Female$_{SR}$ZZ:logMass | -0.08 | -0.42 | 0.31 |
| MaleZZ:logMass | -0.17 | -0.53 | 0.19 |
| *Random Effects* | | | |
| Lizard Identity (id) | | | |
| **Intercept** | **0.18** | **0.11** | **0.26** |
| **Slope** | **0.11** | **0.01** | **0.23** |
| Sampling Session (day) | | | |
| **Intercept** | **0.22** | **0.13** | **0.35** |
| Residuals | | | |
| **Sigma_Intercept** | **-1.67** | **-2.01** | **-1.39** |
| **Sigma_logMass** | **-1.51** | **-2.35** | **-0.53** |
| Sigma_ztime | -0.11 | -0.39 | 0.15 |

**Table S3.** BRMS model coefficients for each respective species when testing mass differences across sex class for animals used in respirometry experiments. Mass was log-transformed and lower and upper bounds were derived from the 95% credible interval for each parameter, estimated from the posterior samples.

| Species | Sex | n | Estimate | l-95% CI | u-95% CI | Contrast | pMCMC Value |
|---|---|---|---|---|---|---|---|
| *B. duperreyi* | Female XX | 15 | 0.04 | -0.02 | 0.11 | Female XX-Male$_{SR}$ XX | 0.45 |
| | Male$_{SR}$ XX | 13 | -0.02 | -0.10 | 0.05 | Male$_{SR}$ XX-Male XY | 0.91 |
| | Male XY | 12 | 0.02 | -0.06 | 0.09 | Male XY-Female XX | 0.70 |
| *P. vitticeps* | Female ZW | 30 | 0.01 | -0.10 | 0.11 | Female ZW-Female$_{SR}$ ZZ | 0.48 |
| | Female$_{SR}$ ZZ | 28 | 0.06 | -0.04 | 0.16 | Female$_{SR}$ ZZ-Male ZZ | 0.44 |
| | Male ZZ | 38 | 0.01 | -0.08 | 0.10 | Male ZZ-Female ZW | 0.96 |

Journal of Experimental Biology • Supplementary information

**Table S4.** BRMS Model coefficients for SVL and mass growth rate estimates across sex class and metabolism for *Bassiana duperreyi* and *Pogona vitticeps*. Growth rate was calculated by dividing the change in growth (SVL or mass) between the initial measurement and subsequent remeasurement by the total number of days elapsed. Due to the small size and rate of change in grams, mass was converted to centigrams for *B. duperreyi* (cg). Animals were remeasured between 3 and 6 months post hatch. Metabolism was estimated by the mean log O2 measurement for each individual.

| Species | Growth rate | Covariate | Estimate | l-95% CI | u-95% CI |
|---|---|---|---|---|---|
| *B. duperreyi* | *SVL (mm/d)* | Intercept (O2_SexFemaleXX) | -4.62 | -5.02 | -4.21 |
| | | Growth Rate (SVLmm) | 6.20 | -2.26 | 14.35 |
| | | O2_SexMaleSRXX | -0.14 | -0.73 | 0.42 |
| | | O2_SexMaleXY | 0.10 | -0.44 | 0.64 |
| | | Growth Rate (SVLmm):O2_SexMaleSRXX | -4.07 | -16.08 | 7.37 |
| | | Growth Rate (SVLmm):O2_SexMaleXY | -7.84 | -19.83 | 4.18 |
| | *Mass (cg/d)* | Intercept (O2_SexFemaleXX) | -4.61 | -4.97 | -4.24 |
| | | Growth Rate (mass cg/d) | 1.06 | -0.98 | 3.04 |
| | | O2_SexMaleSRXX | 0.20 | -0.47 | 0.87 |
| | | O2_SexMaleXY | 0.18 | -0.45 | 0.82 |
| | | Growth Rate (mass cg/d):O2_SexMaleSRXX | -2.59 | -6.49 | 1.25 |
| | | Intercept (O2_SexFemaleXX) | -4.61 | -4.97 | -4.24 |
| *P. vitticeps* | *SVL (mm/d)* | Intercept (O2_SexFemaleZW) | -2.17 | -2.77 | -1.57 |
| | | Growth Rate (SVLmm) | 1.47 | -0.84 | 3.79 |
| | | O2_SexFemaleSRZZ | 0.17 | -0.63 | 0.95 |
| | | O2_SexMaleZZ | 0.01 | -0.73 | 0.74 |
| | | Growth Rate (SVLmm):O2_SexFemaleSRZZ | -1.51 | -4.74 | 1.66 |
| | | Growth Rate (SVLmm):O2_SexMaleZZ | -0.35 | -3.11 | 2.45 |
| | *Mass (g/d)* | Intercept (O2_SexFemaleZW) | -1.79 | -2.30 | -1.27 |
| | | Growth Rate (mass g/d) | -0.07 | -2.55 | 2.41 |
| | | O2_SexFemaleSRZZ | 0.00 | -0.77 | 0.75 |
| | | O2_SexMaleZZ | -0.27 | -0.91 | 0.37 |
| | | Growth Rate (mass g/d):O2_SexFemaleSRZZ | -0.90 | -4.48 | 2.78 |
| | | Growth Rate (mass g/d):O2_SexMaleZZ | 0.92 | -2.08 | 3.83 |

Journal of Experimental Biology • Supplementary information
